# CAR T‐Cell Therapy in Neurology: A Scoping Review of Neuro‐Oncology, Autoimmune Diseases & Neurotoxicity

**DOI:** 10.1002/acn3.70415

**Published:** 2026-04-27

**Authors:** Omar Alqaisi, Mohammed Dibas, Patricia Tai, Ala Dibas, Osama Souied, Suhair Al‐Ghabeesh

**Affiliations:** ^1^ Nursing Department Al‐Zaytoonah University Amman Jordan; ^2^ Department of Medicine, Faculty of Medicine and Health Sciences An‐Najah National University Nablus Palestine; ^3^ Department of Oncology University of Saskatchewan Saskatoon Saskatchewan Canada; ^4^ Roswell Park Comprehensive Cancer Center Buffalo New York USA

**Keywords:** autoimmune neurological disorders, CAR T‐cell, glioblastoma, ICANS, multiple sclerosis, neurology, neurotoxicity, NMOSD

## Abstract

Chimeric antigen receptor (CAR) T‐cell therapy has been investigated in neurological diseases, encompassing both central nervous system malignancies and autoimmune disorders, thereby extending its application beyond hematological cancers. This scoping review evaluates CAR T‐cell therapy applications in neurological conditions, assessing therapeutic efficacy, safety profiles, and neurotoxicity management strategies. A literature search across four databases (January 2020–December 2025) identified 33 studies meeting the inclusion criteria, encompassing original and secondary research from international centers. CAR T‐cell therapy demonstrated promising efficacy across diverse neurological conditions. In glioblastoma trials, 44% of patients (*n* = 128) achieved partial or complete clinical/radiographic responses with favorable safety profiles. Moreover, compelling results emerged from neuromyelitis optica spectrum disorder studies, in which 92% of patients (11/12) achieved sustained relapse‐free remission over a median follow‐up of 5.5 months. Multiple sclerosis, myasthenia gravis, and stiff‐person syndrome cases exhibited excellent treatment tolerance without significant immune effector cell‐associated neurotoxicity syndrome (ICANS), which is a major concern affecting 27% of patients with hematological malignancies. Overall, CAR T‐cell therapy emerges as a novel therapeutic strategy in neurology, encompassing both oncological and autoimmune conditions. Toxicity profiles in neurological CAR T‐cell applications differ substantially from those observed in hematologic malignancies, underscoring the need for condition‐specific risk assessment frameworks and customized management approaches. Future research should prioritize larger multicenter trials with extended follow‐up to establish definitive efficacy and safety profiles in neurological indications.

**Abbreviations**
9‐HPT: Nine‐hole peg test
AChR: acetylcholine receptor
AE: adverse event
AQP4‐IgG: aquaporin‐4 immunoglobulin G
BBB: blood–brain barrier
BCMA: b‐cell maturation antigen
CAR: chimeric antigen receptor
CIDP: chronic inflammatory demyelinating polyneuropathy
CNS: central nervous system
CR: complete response
CRES: CAR T‐related encephalopathy syndrome
CRS: cytokine release syndrome
CSF: cerebrospinal fluid
DAGLA: diacylglycerol lipase alpha
DLTs: dose‐limiting toxicities
EDSS: expanded Disability Status Scale
EEG: electroencephalography
FDA: food and drug administration
ICANS: immune effector cell‐associated neurotoxicity syndrome
IVIG: intravenous immunoglobulin
LICATS: local immune effector cell‐associated toxicity syndrome
MG: myasthenia gravis
MG‐ADL: myasthenia gravis activities of daily living
mRANO/iRANO criteria: modified or immunotherapy response assessment in neuro‐oncology
MRI: magnetic resonance imaging
MS: multiple sclerosis
NfL: neurofilament light chain
NMOSD: neuromyelitis optica spectrum disorder
ORR: objective response rate
OS: overall survival
PCNSL: primary central nervous system lymphoma
PFS: progression‐free survival
PML: progressive multifocal leukoencephalopathy
PR: partial response
QMG: quantitative myasthenia gravis
scFv: single‐chain variable fragment
T25FW: timed 25‐ft walk
TRAE: treatment‐related adverse event

## 1 Introduction

CAR (chimeric antigen receptor) T‐cell therapy has revolutionized adoptive immunotherapy. A patient's T‐cells are engineered to recognize tumor‐associated antigens expressed on cancer cells [1]. The CAR is a synthetic receptor with three essential domains: an extracellular binding domain, typically a single‐chain variable fragment of an antibody (scFv), a hinge and transmembrane domain, and an intracellular domain, which is generally the CD3ζ chain alongside co‐stimulatory domains such as CD28 or 4‐1BB [2]. It has provided remarkable remissions in hematological malignancies, prompting the Food and Drug Administration (FDA) approval of seven CAR T‐cell products targeting CD19 or B‐cell maturation antigen (BCMA) [3]. While it was first developed and approved for hematological malignancies, this review specifically examines CAR T‐cell therapy through a neurological lens, focusing on central nervous system (CNS) tumors and autoimmune neurological diseases in which the CNS or peripheral nervous system is the primary target. Within neurology, oncologic and autoimmune applications of CAR T‐cell therapy are linked by their shared goal of modulating the immune system in or around the CNS.

The most reported immune‐mediated toxicities are cytokine release syndrome (CRS) and CAR T‐related encephalopathy syndrome (CRES), which is now more broadly recognized as immune effector cell‐associated neurotoxicity syndrome (ICANS) [4, 5]. CRS typically develops within a week of infusion and manifests as a systemic inflammatory response characterized by flu‐like symptoms, hypotension, and organ dysfunction [4].

ICANS frequently follows or overlaps with CRS, typically occurring 3–10 days post‐infusion, characterized by encephalopathy, including mild confusion, attention deficits, tremor, handwriting or word‐finding difficulties, and usually mild headaches. In severe cases, this may progress to seizures (1%–30%, mostly tonic–clonic) and coma [5]. Expressive dysphasia progressing to receptive dysphasia is common. The most life‐threatening complication is cerebral edema, particularly associated with CD19‐directed CAR T‐cell therapy. Electroencephalography (EEG) often demonstrates diffuse slowing, while magnetic resonance imaging (MRI) is usually normal but can reveal diffuse cerebral edema in severe cases [5]. A meta‐analysis estimates the incidence of ICANS totaling 27%, with high‐grade events occurring in 10% of patients [6]. CAR T‐cell neurotoxicity is relatively common and can be life‐threatening [6], thus highlighting the need to investigate underlying mechanisms, risk factors, and management strategies in this review.

Another adverse event (AE) is the on‐target/off‐tumor toxicity causing B‐cell aplasia [7]. Since many autoimmune neurological disorders are driven by pathogenic B or T cells, they represent rational targets for CAR T‐cells [7]. These disorders impose a substantial health burden: e.g., multiple sclerosis (MS) affects 2.8 million people worldwide [8]. Other disorders such as neuromyelitis optica spectrum disorder (NMOSD) and autoimmune encephalitis are much rarer, with only a few cases per 100,000, but carry high morbidity and mortality [9, 10]. Early clinical reports are promising. In two patients with treatment‐refractory progressive MS, the fully human anti‐CD19 CAR T‐cell product (KYV‐101) was well tolerated and reduced intrathecal antibodies without neurotoxicities [11]. In NMOSD, a phase I trial of BCMA‐targeted CAR T‐cell product (CT103A) in aquaporin‐4 immunoglobulin G (AQP4‐IgG) seropositive patients achieved sustained remission in 11/12 participants over a median follow‐up of 5.5 months. AEs were limited to grade 1–2 CRS [10]. Similarly, a 69‐year‐old woman with severe treatment‐refractory stiff‐person syndrome experienced marked clinical improvement [12].

CAR T‐cell therapy is now being explored in solid tumors of the central nervous system (CNS), most notably glioblastoma [13]. The underlying rationale is that CAR T‐cells can be engineered to selectively recognize and eliminate glioma‐associated antigens within the CNS [14]. Multiple phase I trials evaluating CAR T‐cell therapies target EGFRvIII, IL13Rα2 and HER2 [14]. A systematic review of 13 early glioblastoma trials (128 patients) noted a 44% radiographic or clinical response rate. Importantly, most studies confirmed the safety of intracranial delivery, with doses administered up to 2.5 × 10^7^ with only 2 dose‐limiting toxicities (DLTs) [15].

This review covers the clinical applications of CAR T‐cell therapy in neurological diseases (autoimmune neurologic conditions and cancers) and neurotoxicities. Ongoing challenges and gaps will be highlighted throughout this review.

## 2 Methods

This review was conducted according to a predefined protocol based on the framework proposed by Arksey and O'Malley, the Preferred Reporting Items for Systematic Reviews and Meta‐Analyses extension for Scoping Reviews (PRISMA‐ScR) [16] and the Population‐Concept‐Context framework [17]. The protocol was developed in advance but not registered, as PROSPERO does not currently accept scoping review protocols.

Electronic databases (PubMed, Scopus, Embase, and Web of Science) were searched from January 1, 2020, to December 10, 2025. This timeframe was selected to capture the most recent and clinically relevant phase of research in neurological applications of CAR T‐cell therapy, a field that remains at an early stage of clinical development. The research question was “What evidence exists regarding the efficacy, safety, and neurotoxicity management of CAR T‐cell therapy in adult patients with CNS malignancies and autoimmune neurological disorders?”

Search strategies utilized a combination of Medical Subject Headings (MeSH) and free text terms including: (“CAR T‐cell*” OR “chimeric antigen receptor T‐cell*” OR “CAR‐T" OR “CAR T therapy” OR “CAR‐T therapy” OR “CAR T lymphocyte*” OR “CAR‐T immunotherapy” OR “chimeric antigen receptor T therapy” OR “CAR T‐cell therapy” OR “CAR T cells”) AND (“neurology*” OR “neurological” OR “CNS” OR “central nervous system” OR “brain tumor*” OR “glioma*” OR “glioblastoma” OR “CNS lymphoma” OR “primary CNS lymphoma” OR “PCNSL” OR “autoimmune*” OR “immune‐mediated” OR “multiple sclerosis” OR “MS” OR “NMOSD” OR “neuromyelitis optica” OR “stiff‐person syndrome” OR “myasthenia gravis”) AND (“ICANS” OR “neurotoxicity” OR “neurotoxic*” OR “encephalopathy” OR “CRES” OR “Adverse Effects”) Database‐specific strategies with applied filters are provided in Table S1.

### 2.1 Eligibility Criteria

#### 2.1.1 Inclusion Criteria

Studies were eligible for inclusion if they met all of the following:

- **Population:** Adult human participants (≥ 18 years) receiving CAR T‐cell therapy for central nervous system malignancies (e.g., glioblastoma, CNS lymphoma, CNS involvement of systemic hematologic malignancies), autoimmune neurological diseases (e.g., multiple sclerosis, neuromyelitis optica spectrum disorder, myasthenia gravis, stiff‐person syndrome, chronic inflammatory demyelinating polyneuropathy, autoimmune encephalitis), or adult CAR T‐cell recipients in whom neurotoxicity or neurological outcomes were specifically reported.
- **Concept:** Studies reporting at least one of the following: therapeutic efficacy (e.g., radiographic or clinical response, relapse‐free remission, progression‐free survival, or overall survival), safety outcomes (e.g., incidence and grading of cytokine release syndrome or ICANS), or characterization and/or management of CAR T‐cell–related neurotoxicity.
- **Context:** Peer‐reviewed articles published in English between January 1, 2020 and December 10, 2025, indexed in PubMed, Scopus, Embase, or Web of Science, using any of the following designs: phase I–III clinical trials, prospective or retrospective cohort studies, case reports/series, or systematic/narrative reviews.

#### 2.1.2 Exclusion Criteria

Studies were excluded if they were (1) editorials, commentaries, conference abstracts, letters without original data, or narrative perspectives without primary or systematically synthesized evidence; (2) preclinical, animal, or in vitro studies; (3) pediatric populations (< 18 years); (4) non‐English‐language publications; or (5) duplicate reports of the same cohort, in which case the most complete or recent publication was retained.

As a scoping review, the methodological objective differs from that of a systematic review. According to the PRISMA‐ScR guidelines, scoping reviews aim to map the breadth and nature of existing evidence across heterogeneous study designs, including both primary research and the secondary literature (systematic/narrative reviews) for comprehensive evidence mapping and identification of consistent patterns across varied settings.

**FIGURE 1:**
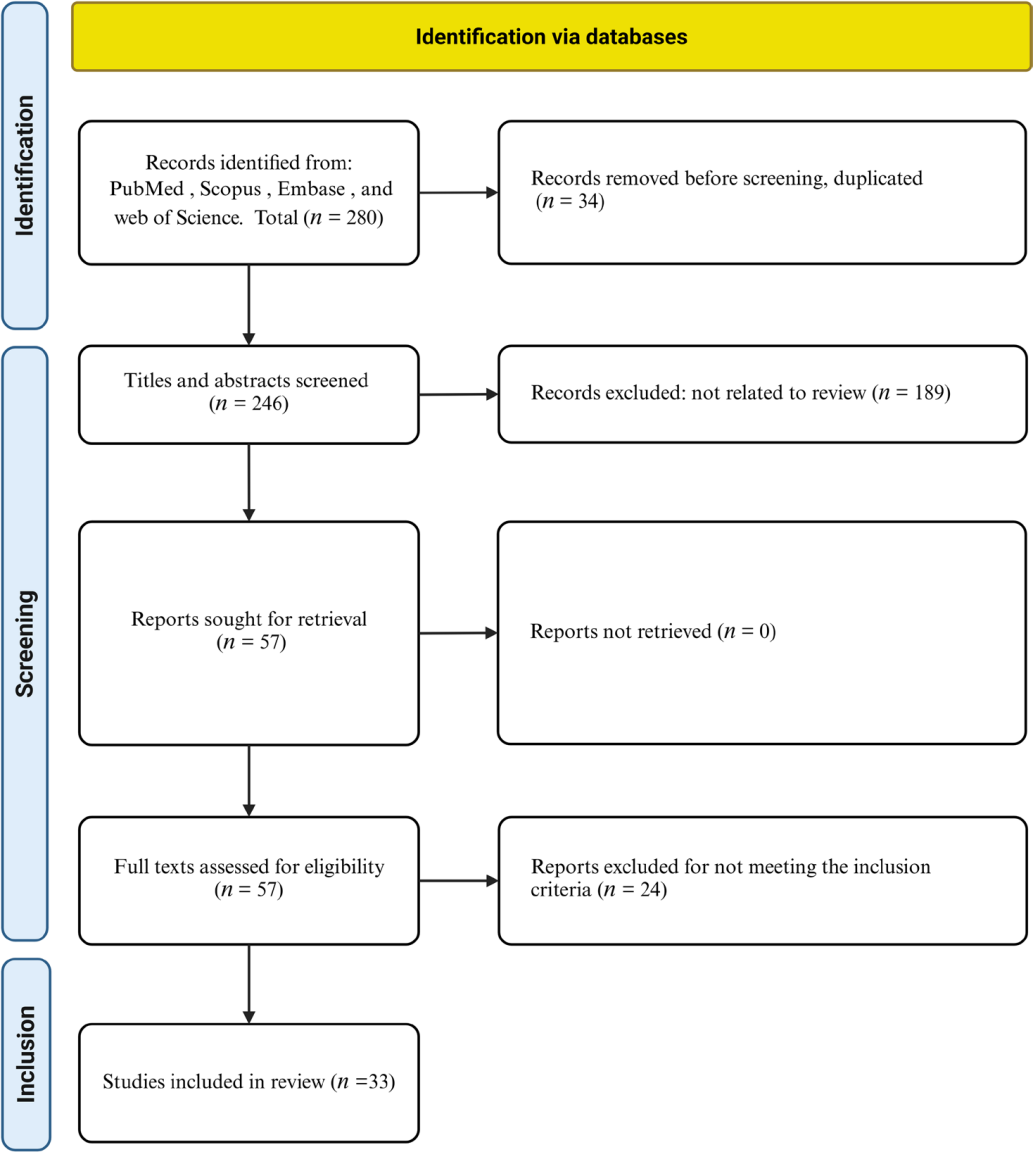
Preferred Reporting Items for Systematic Reviews and Meta‐Analyses extension for Scoping Reviews (PRISMA‐ScR) flow diagram of the study selection process.

All identified citations were imported into citation management software, and duplicates were removed. Two independent authors (O.A., M.D.) screened titles and abstracts against the eligibility criteria. Full‐text articles of potentially relevant studies were retrieved and assessed for inclusion. Any disagreement was resolved through consultation with a third author (A.D.) (Figure 1). Ultimately, **33** articles met the inclusion criteria.

Data were extracted on key study characteristics, including author, objective, CAR T‐cell product, study design, sample size, and key outcomes (efficacy, safety, and neurotoxicity), as detailed in Table S2 [6, 10, 11, 12, 15, 18, 19, 20, 21, 22, 23, 24, 25, 26, 27, 28, 29, 30, 31, 32, 33, 34, 35, 36, 37, 38, 39, 40, 41, 42, 43, 44, 45, 46]. The PRISMA‐ScR checklist is provided in Table S3.

## 3 Results

The 33 studies employed various research designs: case reports (*n* = 6), systematic reviews (*n* = 5), literature reviews (*n* = 10), phase I clinical trials (*n* = 4), phase II clinical trials (*n* = 2), retrospective cohorts (*n* = 5), and prospective cohorts (*n* = 1). They originated from multiple countries, including Canada (*n* = 2), Korea (*n* = 1), Germany (*n* = 6), China (*n* = 9), the United Kingdom (*n* = 4), Switzerland (*n* = 2), the United States (*n* = 6), Mexico (*n* = 1), and multinational studies (*n* = 2), reflecting diverse geographical perspectives on CAR T‐cell therapy applications in neurology.

### 3.1 CAR T‐Cell Therapy in CNS Malignancies

CAR T‐cells have emerged as a promising therapeutic modality for CNS malignancies, particularly in treatment‐refractory settings. An analysis of 13 phase I clinical trials demonstrated promising efficacy for CAR T‐cells in glioblastoma [15]. Across 128 patients treated in these early‐phase trials, 44% demonstrated partial radiographic responses on serial MRI scans using mRANO/iRANO criteria (Modified or Immunotherapy Response Assessment in Neuro‐Oncology) that included initial tumor necrosis and, in some cases, reduced cerebrospinal fluid (CSF) antigen levels. Median overall survival (OS) following infusion ranged from 2.9–14.5 months, while progression‐free survival (PFS) ranged from 1.3–5.5 months. Across the analyzed studies, six tumor‐associated antigens were targeted: EGFRvIII, IL13Rα2, HER2, GD2, EphA2, and PD‐L1, with the first two being the most common. None of the investigated antigen targets demonstrated clear superiority in clinical outcomes, as all showed transient antigen reduction followed by escape. Specific antigen targets showed differing toxicity profiles; anti‐IL13Rα2 trials appeared to show more neurological AEs, while anti‐EGFRvIII trials trended toward more hematological toxicities. Most studies demonstrated reduced target antigen expression post‐infusion (detected via tumor biopsies and CSF analysis), without any correlation with clinical benefit. Administration route has been explored in trials without a clear difference in efficacy. Locoregional delivery (intraventricular or intratumoral) achieved higher levels in CSF compared to intravenous infusion, without consistent clinical benefits in safety or outcome. There is no clear dose–response relationship with doses from 10^6^ to 10^10^ cells nor evidence of DLTs, though respiratory failure and fatigue occurred in two patients at doses exceeding 2.5 × 10^7^ cells. CAR T‐cell products exhibited poor persistence, peaking at days 7–14 post‐infusion before becoming undetectable by 1 month, accompanied by T‐cell exhaustion marked by PD‐1 and LAG‐3 upregulation.

In a retrospective multicenter study of 54 adults with relapsed/refractory (R/R) CNS lymphoma (including primary and secondary cases) [32], at 100 days post‐infusion, 65% of patients achieved a complete response (CR). However, the median PFS/OS were 7.5 and 19 months, respectively, and the 1‐year OS rate was 63%. Interestingly, different CAR T‐cell products had variable outcomes: tisagenlecleucel demonstrated significantly shorter PFS and OS versus axicabtagene ciloleucel (axi‐cel), whereas lisocabtagene maraleucel demonstrated improved survival relative to axi‐cel. Importantly, no new safety signals emerged: grade ≥ 3 CRS in 11% and grade ≥ 3 ICANS in 28% of patients. Two patients (4%) experienced fatal treatment‐related AEs (TRAEs). Active CNS disease did not significantly increase neurotoxicity risk.

Following the success of second‐generation CAR T‐cell therapy in CNS lymphoma [32], it has now been extended to secondary CNS malignancies, with a phase II study of third‐generation CD19 CAR T‐cells in 21 patients with R/R B‐cell malignancies and CNS involvement, achieving a 71% objective response rate (ORR) and median duration of response of 11.1 months [36]. At a median follow‐up of 20.4 months, the 12‐month PFS and OS were 41.5% and 61.2%, respectively. Manageable ICANS occurred in 43%, including 29% with grade ≥ 3 events.

For the challenging CNS involvement in multiple myeloma, emerging evidence of CAR T‐cell therapy directed against BCMA was reported: 10 patients with R/R disease received either idecabtagene vicleucel or ciltacabtagene autoleucel [33]. Treatment in this high‐risk group was feasible and safe, with no grade ≥ 3 CRS and few ICANS (no grade 4, one grade 3, two grade 1). ORR was 80%, though responses were short‐lived (PFS 6.3 months; OS 13.3 months). Optimized CNS‐directed bridging therapy (systemic or intrathecal chemotherapy ± radiotherapy) achieved better outcomes. There may be a need for consolidation or maintenance strategies.

### 3.2 CAR T‐Cell Therapy in Autoimmune Neurological Disorders

The most robust evidence comes from a phase I open‐label trial of anti‐BCMA CAR T‐cells in 12 patients with R/R aquaporin‐4 immunoglobulin G (AQP4‐IgG) seropositive NMOSD [10]. Over a median follow‐up of 5.5 months, 11/12 patients achieved sustained remission, with marked reductions in pathogenic AQP4‐IgG antibody titers. A companion study used single‐cell multi‐omics analysis of CSF and blood from five NMOSD patients, showing anti‐BCMA CAR T‐cells efficiently crossed the Blood–brain barrier (BBB) [28]. High expression of the chemotaxis receptor CXCR3 was correlated with enhanced CNS infiltration and therapeutic efficacy.

The first clinical application of a fully human anti‐CD19 CAR T‐cell product (KYV‐101) was reported in two patients with treatment‐refractory progressive MS demonstrating excellent treatment tolerance without AEs [11]. Also, a phase I trial NCT04561557 enrolled five patients with refractory progressive MS [37, 38] for a single intravenous infusion of equecabtagene autoleucel (Eque‐cel), a fully human anti‐BCMA CAR T‐cell therapy, administered at a dose of 1 × 10^6^ cells/kg following lymphodepleting chemotherapy. CAR T‐cells rapidly reached peak expansion around day 10, and remained detectable in blood, CSF, and bone marrow for up to 3 months. All five patients improved significantly on the Expanded Disability Status Scale (EDSS), nine‐hole peg test (9‐HPT), and timed 25‐ft walk (T25FW). In addition, follow‐up MRI scans revealed no new T1 gadolinium‐enhancing lesions or new/enlarging T2 lesions, accompanied by a complete resolution of CSF oligoclonal bands. Treatment was safe, with transient grade 1 CRS in 80% of patients and no grade ≥ 2 CRS or neurotoxic reactions. All grade ≥ 3 cytopenias occurred within 40 days, without grade ≥ 3 anemia or thrombocytopenia. Preclinical and early clinical evidence across six trials (NCT04561557, NCT06138132, NCT06220201, NCT06249438, NCT06384976, NCT06451159) highlights a favorable safety profile in ongoing phase I/II trials targeting progressive and treatment‐refractory scenarios [41]. A single infusion of anti‐CD19 or BCMA CAR T‐cells showed no high‐grade CRS, maintaining clinical stability over 100 days without early neurotoxicity. While neurotoxicities such as delayed parkinsonian syndromes or progressive multifocal leukoencephalopathy (PML) remain concerns, biomarkers such as serum neurofilament light chain (NfL) and plasma brain‐derived cell‐free DNA (bcfDNA) may aid in early detection and intervention, although widespread clinical implementation is still preliminary.

For other autoimmune neurological conditions, a 69‐year‐old woman with severe treatment‐refractory stiff‐person syndrome experienced markedly reduced muscle stiffness and increased ambulatory capacity, with low‐grade CRS [12]. In addition, two patients with R/R chronic inflammatory demyelinating polyneuropathy (CIDP) received BCMA‐directed CAR T‐cell therapy and achieved rapid drug‐free remission within 6 months, without severe AEs [29]. One patient remained in sustained remission for over 2 years at last follow‐up, whereas the other relapsed at 12 months, coinciding with a severe COVID‐19 infection.

For myasthenia gravis (MG), the first phase Ib/IIa trial (MG‐001, NCT04146051) of Descartes‐08, an RNA‐engineered anti‐BCMA CAR T‐cell therapy administered without lymphodepletion, demonstrated safety and efficacy in 14 adults with generalized MG. By week 12, mean reductions from baseline were 5.9 points in MG‐Activities of Daily Living (MG‐ADL) and 7 points in Quantitative MG (QMG). There was a modest anti‐AChR titer drop. All patients exceeded established thresholds for clinically meaningful improvement, with two discontinuing Intravenous immunoglobulins (IVIG), without DLTs/CRS/ICANS/grade ≥ 3 events. These effects persisted for up to 9 months [39]. Another study (single‐arm, phase I dose‐escalation trial of anti‐BCMA/CD19 bispecific CAR T‐cells) of 18 adults with refractory generalized MG (all with baseline MG‐ADL scores ≥ 6 and QMG scores ≥ 8) showed no significant toxicities across this dose range (1.0–5.0 × 10^6^/kg). At 180‐day follow‐up, 100% achieved ≥ 2‐point MG‐ADL and ≥ 3‐point QMG reductions, 88% discontinued glucocorticoids and all stopped non‐steroidal immunosuppressants. Nearly half of patients with detectable anti‐AChR antibodies at baseline lost seropositivity. Circulating B cells were depleted by day 7 (61%), with recovery observed in 56% of patients by day 180 [40].

For other rare, severe CNS autoimmune disorders, a 36‐year‐old man with treatment‐refractory autoimmune encephalitis associated with diacylglycerol lipase alpha (DAGLA) antibodies [35] received a single infusion of an experimental fully human CD19 CAR T‐cell product (KYV‐101) after failing multiple prior treatments, including corticosteroids, plasma exchange, and B‐cell depletion with rituximab. His rapid, sustained improvement included loss of anti‐DAGLA autoantibodies in serum and CSF and clearance of CSF oligoclonal bands. No serious AEs were observed, even in the setting of active resistant severe encephalitis.

### 3.3 Neurotoxicity Profile and Safety Considerations of CAR T‐Cell Therapy

A meta‐analysis incorporating 2592 patients across 84 clinical trials reported a 40% incidence of neurotoxicities, with 28% being grade ≥ 3, occurring more frequently in hematological malignancies than in solid tumors [24]. Another meta‐analysis of 3184 patients reported an ICANS incidence of 26.9%, with 10.5% experiencing grade ≥ 3 toxicity [6]. ICANS risk was higher with anti‐CD19 CAR T‐cells than with anti‐BCMA constructs, being highest in leukemia. Higher doses, age > 65 years, preexisting neurological abnormalities, and early/severe CRS are associated with higher ICANS incidence. ICANS occurred much more frequently with axicabtagene ciloleucel than with tisagenlecleucel. All reported ICANS‐related deaths were linked to axicabtagene ciloleucel, and this higher incidence and severity has been correlated with the CD28 costimulatory endodomain present in axicabtagene ciloleucel and brexucabtagene autoleucel compared to 4‐1BB endodomain products such as tisagenlecleucel and lisocabtagene maraleucel [6, 43].

ICANS typically starts around day 5 post‐infusion, peaking around days 7–8, usually monophasic and resolves in < 2 weeks with treatment but can rarely be delayed (> 3 weeks) [43]. Diagnosis relies on the Immune effector Cell‐associated Encephalopathy (ICE) score, a bedside cognitive scoring system that evaluates orientation (4 points), naming three objects (3 points), following a simple command (1 point), writing a standard sentence (1 point), and attention by counting backward from 100 in tens (1 point). This score is incorporated into the American Society for Transplantation and Cellular Therapy (ASTCT) consensus holistic grading, which incorporates consciousness, seizures, motor weakness, and imaging features, to classify ICANS as low‐grade (1–2) vs. high‐grade (≥ 3) [21, 43]. Most included studies reporting ICANS used ICE‐based or ASTCT‐aligned grading, although the level of reporting detail was not fully uniform across all studies. Neuroimaging is often normal, whereas EEG typically demonstrates diffuse slowing (97.2%) and triphasic waves (41.7%), with no electrographic seizures detected despite clinical manifestations [31, 43]. ICANS management varies by grade: grade 1 requires conservative monitoring only, while higher grades (2–4) necessitate corticosteroids (grades 2–3: dexamethasone 10 mg IV every 6–12 h; grade 4: methylprednisolone 1 g IV daily for 3 days, with rapid taper) [20, 30, 43]. Early steroid use has been shown to reduce severe ICANS rates. Tocilizumab, an IL‐6 receptor blocker, can be used as an adjunct to corticosteroids for ICANS management when concurrent CRS is present. Additionally, seizure prophylaxis with levetiracetam is recommended for patients with ICANS grade ≥ 1. Options for steroid‐refractory cases include anakinra (high‐dose > 200 mg/day) or intrathecal chemotherapy (steroids/methotrexate/cytarabine). Recently, siltuximab, a monoclonal antibody that directly binds circulating IL‐6, demonstrated promising efficacy in managing CRS and ICANS in a multicenter retrospective study, with an ORR of 75% for CRS (median resolution 1 day) and 60% for ICANS (median resolution 4.5 days) [34]. Notably, it also appeared effective in patients with CRS previously exposed to tocilizumab, with an ORR of 63%. A possible advantage of siltuximab in neurotoxicity lies in its mechanism: while tocilizumab's receptor blockade may increase circulating IL‐6, siltuximab neutralizes IL‐6 directly and may avoid this effect. At present, siltuximab may be considered in selected cases of tocilizumab‐refractory CRS, supported by preliminary clinical efficacy data. Nevertheless, current evidence remains insufficient to draw firm conclusions regarding its comparative efficacy or safety relative to other treatments.

Multiple studies point to a common cascade initiated by cytokine release in conjunction with immune overactivation leading to endothelial insult and disruption of the BBB [18, 21, 22, 26]. Cytokines such as IL‐1, IL‐6, IL‐15, GM‐CSF, and TNF‐α contribute to astrocyte and microglial activation, promoting CNS inflammation [22, 26]. Various proposed mechanisms include spikes in cytokines, blood vessel leaks, and possible off‐tumor CAR T‐cell effects [21]. In an analysis of 101 CD19 CAR T‐cell recipients, CSF cytokine levels correlated with ICANS severity (grade 2–4), with peak symptoms associated with elevated IL‐6, IL‐15, GM‐CSF, and IL‐1β [46]. Persistent elevations tracked resolution, supporting their utility as neurotoxicity biomarkers.

While ICANS remains the most common neurological complication, over the past 4 years emerging non‐classical neurotoxicities have been reported with newer CAR T‐cell targets: movement and neurocognitive toxicity (MNT), cranial nerve palsies, tumor inflammation‐associated neurotoxicity (TIAN), ischemic stroke, acute/chronic myelitis and cranial neuropathy [42] (Table 1).

**TABLE 1:** Most commonly reported non‐ICANS neurotoxicities in CAR T‐cell therapy recipients.

Toxicity	CAR T‐cell product	Clinical picture	Management
Movement and Neurocognitive Toxicity (MNT)	BCMA‐targeted (e.g., idecabtagene vicleucel, ciltacabtagene autoleucel)	New‐onset movement disorder (tremor, cogwheel rigidity, gait disturbance), cognitive impairment (reduced attention/memory), personality changes (apathy, flat affect); median onset 36 days post‐CRS/ICANS.	1st‐line: Dexamethasone 10–20 mg IV, 2–4 times daily for 3–5 days. 2nd‐line: Cyclophosphamide 2 g/m^2^ IV. 3rd‐line: Etoposide or alternative chemotherapy.
Tumor Inflammation‐Associated Neurotoxicity (TIAN)	CAR T‐cell products in CNS tumors	Tumor location‐dependent, Type 1: ICP ≥ 20 mmHg (headache →herniation) Type 2: transient worsening of pre‐existing symptoms.	Type 1: urgent ICP lowering by CSF diversion, steroids, hyperosmolar fluids, and pre‐emptive Ommaya. Type 2: observation ± steroids/anakinra
Cranial Nerve Palsy	BCMA‐targeted CAR T‐cells (e.g., ciltacabtagene autoleucel), lower incidence with CD19‐directed products.	Predominantly VII palsy (unilateral or bilateral), less frequent involvement of III and V. median onset: 22 days.	Short‐course corticosteroids (e.g., prednisolone 25 mg BID for 10 days), ~90% of cases resolve.
Ischaemic Stroke	Primarily CD19‐targeted CAR T‐cells	Sudden focal deficits (unilateral weakness, facial droop, speech issues, hemianopsia); may be asymptomatic.	Blood pressure management (permissive hypertension), individualized antiplatelets/anticoagulation/thrombolysis.
Peripheral Neuropathy/Guillain‐Barré Syndrome	CD19‐targeted CAR T‐cells (tisagenlecleucel) and BCMA‐targeted (ciltacabtagene autoleucel).	Symmetric ascending weakness, areflexia, sensory changes, back pain, and possible respiratory failure in severe cases.	Steroids + IVIG (5 days).

Abbreviations: BCMA, b‐cell maturation antigen; BID, twice daily; CAR, chimeric antigen receptor; CN, cranial nerve; CNS, central nervous system; CSF, cerebrospinal fluid; ICANS, immune effector cell‐associated neurotoxicity syndrome; ICP, intracranial pressure; IV, intravenous; IVIG, intravenous immunoglobulin; MNT, movement and neurocognitive toxicity; TIAN, tumor inflammation‐associated neurotoxicity.

MNT, most frequently reported with BCMA‐directed CAR T‐cell therapies such as idecabtagene vicleucel and ciltacabtagene autoleucel in multiple myeloma, typically emerges following the resolution of CRS or ICANS [42, 43]. The median onset is approximately 36 days, with parkinsonism‐like symptoms including tremor, cogwheel rigidity, micrographia, gait disturbance, hypophonia, apathy, cognitive impairment, and flat affect. Management includes high‐dose dexamethasone (10–20 mg administered 2–4 times daily for 3–5 days), with escalation to intravenous cyclophosphamide (2 g/m^2^) in refractory cases, followed by etoposide if needed. In contrast to MNT, which typically emerges later, TIAN presents acutely within days of infusion [42, 43]. TIAN is a distinct CAR T‐cell therapy AE observed with CNS solid tumors or hematological malignancies involving the CNS. It is driven by localized tumor‐site inflammation that elevates intracranial pressure (≥ 20 mmHg), with clinical features reflecting the tumor location. TIAN can be classified into two overlapping phenotypes: Type 1 (mechanical, with headache and herniation from mass effect) and Type 2 (inflammatory, with transient worsening of pre‐existing neurological deficits due to inflammation or electrophysiological dysfunction). Neuroimaging shows hydrocephalus in Type 1 and T2 hyperintensities in Type 2. Management of Type 1 requires urgent CSF diversion, corticosteroids, hyperosmolar therapy, and pre‐emptive Ommaya reservoir placement for posterior fossa tumors, whereas Type 2 is often managed with observation, corticosteroids, or anakinra for critical sites like brainstem or spinal cord.

Recent reports also described delayed cranial neuropathies following BCMA CAR T‐cell therapy with ciltacabtagene autoleucel [44, 45]: a 75‐year‐old male developed left facial palsy on day 19, progressing to bilateral facial weakness and left VI palsy by day 31 without ICANS [44]. MRI demonstrated bilateral facial nerve enhancement, with unremarkable CSF analysis. High‐dose intravenous corticosteroids led to VI nerve resolution at 2.5 months and improved facial nerve function, as measured by the House–Brackmann scale, at 5.5 months. Similarly, another 76‐year‐old with bilateral facial palsy 2 weeks post‐infusion had mild CSF lymphocytic pleocytosis (T‐cell predominant), and full resolution by day 42 after dexamethasone [45]. Moreover, cerebellar symptoms have also been reported as part of the delayed neurotoxicity spectrum in some myeloma‐directed CAR T‐cells, although they remain rare and are less well characterized than other delayed neurological toxicities [42].

## 4 Discussion

The integration of CAR T‐cell therapy into the neurological field is both timely and necessary. Autoimmune neurological disorders share mechanisms that make them reasonable candidates for CAR T‐cells, since they selectively target disease‐driving cells [7]. The applications are expanding rapidly, with early evidence supporting their use in glioblastoma [15], B‐cell malignancies with CNS involvement [32, 36], MS [11, 37, 41], NMOSD [10, 28], CIDP [29], stiff‐person syndrome [12], DAGLA antibody–associated autoimmune encephalitis [35], and MG [39, 40]. Notably, patients treated for autoimmune neurological conditions demonstrated a more favorable safety profile compared with those receiving CAR T‐cell therapy for hematologic malignancies. MS, MG, and stiff‐person syndrome showed good tolerance [11, 12, 37, 39, 40], while NMOSD patients only experienced mild CRS without any ICANS [10]. This contrasts sharply with the high rates of severe ICANS and CRS in hematologic malignancies [47]. However, these findings should be interpreted with caution, as they are largely based on case reports and early phase I trials with limited follow‐up. Moreover, a newly recognized toxicity named local immune effector cell‐associated toxicity syndrome (LICATS) has recently been observed in the context of autoimmune diseases [48]. Unlike the diffuse neurotoxicity of ICANS, LICATS manifests as a localized, organ‐specific inflammatory reaction that is typically self‐limited. Although evidence in autoimmune neurological conditions remains limited, the identification of LICATS highlights that adverse‐event profiles in autoimmune CAR T‐cell applications may diverge from those observed in hematologic malignancies.

Overall, the included studies demonstrate emerging applications of CAR T‐cell therapy in autoimmune neurological diseases that are broadly concordant with the EBMT expert‐based recommendations for innovative cellular therapies in autoimmune diseases, which emphasize its use in severe, treatment‐refractory patients alongside a careful risk–benefit assessment rather than early‐line deployment. Across NMOSD, MS, MG, and CIDP, investigators consistently selected adults with active disease despite biologic or second‐line immunosuppressive therapy. Reported studies incorporated many of the EBMT‐recommended assessments including neurological examination, MRI, disability or functional scales, EEG, nerve conduction studies, and CSF or serological biomarkers. Early safety signals were emphasized in these studies, including the low incidence of ICANS and the absence of unexpected CNS events. However, the limited sample sizes, short follow‐up periods, partial implementation of the full EBMT work‐up (including systematic cognitive testing, anticonvulsant prophylaxis for CNS involvement, and standardized long‐term surveillance), and the predominance of single‐arm designs underscore that these studies should be viewed as proof of concept rather than definitive, guideline‐level evidence [49].

To monitor neurotoxicity in CAR T‐cell recipients, close neurological monitoring is essential. Starting from day 1 post‐infusion, clinicians should focus on flu‐like symptoms, hypotension, and hypoxia, which could indicate CRS preceding ICANS [4]. Additionally, conducting daily ICE scoring, alongside checks for tremors or difficulty speaking provides a structured approach to the early detection of ICANS [5, 43]. Beyond day 30, the focus should be directed toward delayed non‐ICANS events, including movement issues and neurocognitive problems such as parkinsonism or apathy, as well as cranial neuropathies [42]. The minimum workup should include ICE scoring at symptom onset, an urgent EEG to rule out seizures, and a brain MRI to check for edema or new lesions [43]. Moreover, CSF analysis can detect cytokine levels and CAR T‐cell persistence while excluding alternative pathologies such as bacterial, viral, or fungal infections [43].

Additionally, although ICANS is now well characterized in hematologic malignancies, its manifestation in patients with pre‐existing CNS pathology warrants particular attention. Pre‐existing neurological comorbidities, including structural CNS disease, may produce overlapping clinical and radiographic features that complicate diagnosis. We urge future trials of CAR T‐cell therapy in neurological indications, such as autoimmune encephalitis, to explore this area in greater depth and to determine whether disease‐specific neurotoxicity frameworks are needed, including predefined criteria to distinguish ICANS from disease flares.

Current clinical evidence supporting CAR T‐cell therapy in neurological diseases remains markedly limited, preventing broad conclusions regarding efficacy and safety. Most available data originate from small phase I safety/dosing trials with small sample sizes, lacking randomized controls, standardized endpoints, or long‐term follow‐up data. Future research must prioritize adequately powered phase II/III randomized controlled trials that establish standardized efficacy endpoints. Critical areas include long‐term safety profiling with a minimum of 3–5 years follow‐up to quantify risks of prolonged B‐cell aplasia‐related infections, secondary malignancies, and distinct neurotoxicity profiles.

Beyond the studies already conducted and included in this review, the field of CAR T‐cell therapy for neurological diseases is expanding rapidly, with multiple ongoing clinical trials registered for a broad range of neurological indications (Table 2).

**TABLE 2:** Registered major ongoing clinical trials of CAR T‐cell therapy in autoimmune neurological diseases.

Trial name	NCT number	Condition(s)	CAR T‐cell target	Phase	Status
KYSA‐8	NCT06588491	Stiff‐person syndrome	Anti‐CD19	Phase II	Active (not recruiting)
KYSA‐6	NCT06193889	Generalized MG	Anti‐CD19 (miv‐cel)	Phase II/III	Recruiting
KYSA‐7	NCT06384976	Progressive MS (PPMS/SPMS)	Anti‐CD19 (miv‐cel)	Phase II	Active (not recruiting)
AURORA	NCT06799247	Generalized MG	Anti‐BCMA (Descartes‐08, mRNA)	Phase III	Recruiting
___	NCT04561557	MS, NMOSD, MG, CIDP, AE	Anti‐BCMA (Eque‐cel)	Phase I	Recruiting
___	NCT06138132	Non‐relapsing progressive MS	Anti‐CD19	Phase I	Ongoing
___	NCT06220201	Relapsing/progressive MS; MG	Anti‐CD19 (BMS‐986353)	Phase I	Recruiting
___	NCT06249438	NMOSD, MS, MG	Bi‐specific CD20/BCMA	Phase I	Recruiting
___	NCT06869278	NMOSD, MOGAD, MS, MG	Tri‐specific CD19/CD20/CD22	Phase I	Recruiting
___	NCT06485232	NMOSD, MG, CIDP, MS	Anti CD19/BCMA	Phase I	Not yet recruiting
___	NCT06451159	Refractory MS	Anti‐CD19	Phase I	Not yet recruiting

Abbreviations: AE, autoimmune encephalitis; CIDP, chronic inflammatory demyelinating polyneuropathy; MG, myasthenia gravis; MOGAD, myelin oligodendrocyte glycoprotein antibody‐associated disease; MS, multiple sclerosis; NMOSD, neuromyelitis optica spectrum disorder; PPMS/SPMS, primary/secondary progressive multiple sclerosis.

Limitation of this review was the restriction to English‐language peer‐reviewed studies, which may have reduced comprehensiveness by excluding relevant non‐English publications. However, given that the evidence for CAR T‐cell therapy in neurology remains limited and recent, this restriction is unlikely to have substantially changed the overall direction of the findings. Additionally, most included clinical trials were early phase (phase I), featured short follow‐up periods, and had small sample sizes, limiting generalizability and long‐term insights.

## 5 Conclusions

This scoping review highlights CAR T‐cell therapy's real potential in neurology. In glioblastoma, a 44% response rate via safe intracranial administration was reported, though the benefits did not last long. Furthermore, NMOSD showed 92% relapse‐free remission with minimal neurotoxicity alongside encouraging early efficacy signals in MS, MG, stiff‐person syndrome, and CIDP. Moreover, minimal or no ICANS occurred in autoimmune neurological cohorts, indicating a safer profile compared to hematologic malignancies, where meta‐analyses reported ICANS in ~27% overall, 10% high‐grade, and neurotoxicity in 40% of patients, rates far exceeding those seen in autoimmune neurological diseases. Improved understanding of ICANS pathophysiology including cytokine release, endothelial dysfunction, and BBB disruption has enabled targeted management with corticosteroids, siltuximab, anakinra, and emerging strategies. Overall, CAR T‐cell therapy shows promise in neurological diseases but requires careful clinical development before widespread adoption, as most trials are Phase I with small samples. Future research must prioritize larger multi‐center Phase II/III trials with extended follow‐up.

## Author Contributions

Omar Alqaisi and Mohammed Dibas: conceptualization, methodology, data curation, formal analysis, writing the original draft, writing‐review. Patricia Tai: validation, supervision, writing‐review and editing. Ala Dibas: data curation, validation and writing‐review. Osama Souied and Suhair Al‐Ghabeesh: supervision, writing‐review and editing. All authors read and approved the final manuscript.

## Funding

The authors have nothing to report.

## Ethics Statement

This study did not require approval from an ethics committee, as it involved no primary patient data and was based exclusively on previously published, anonymized data.

## Consent

The authors have nothing to report.

## Conflicts of Interest

The authors declare no conflicts of interest.

## Supporting information


**Table S1:** The detailed search strategy.
**Table S2:** Summary of included 33 studies.
**Table S3:** Preferred Reporting Items for Systematic reviews and Meta‐Analyses extension for Scoping Reviews (PRISMA‐ScR) Checklist.

## Data Availability

The authors have nothing to report.
