# Supplementary material for: CAR T‐Cell Therapy in Neurology: A Scoping Review of Neuro‐Oncology, Autoimmune Diseases & Neurotoxicity
Source: Ann Clin Transl Neurol. 2026 Apr 27;13(7):1310–9. doi: 10.1002/acn3.70415 (PMC13358562; doi:10.1002/acn3.70415)
Supplement: Supplementary file 1 — Table S1: The detailed search strategy. Table S2: Summary of included 33 studies. Table S3: Preferred Reporting Items for Systematic reviews and Meta‐Analyses extension for Scoping Reviews (PRISMA‐ScR) Checklist. [file ACN3-13-1310-s001.docx]

**Table S1**. The detailed search strategy

| **Database** | **Search Strategy** | **Records Retrieved** |
| --- | --- | --- |
| PubMed | Search Terms:  (“CAR T-cell*”[Title/Abstract] OR “chimeric antigen receptor T-cell*”[Title/Abstract] OR “CAR-T”[Title/Abstract] OR “CAR T therapy”[Title/Abstract] OR “CAR-T therapy”[Title/Abstract] OR “CAR T lymphocyte*”[Title/Abstract] OR “CAR-T immunotherapy”[Title/Abstract] OR “chimeric antigen receptor T therapy”[Title/Abstract] OR “CAR T-cell therapy”[Title/Abstract] OR “CAR T cells”[Title/Abstract]) AND (“neurology*”[Title/Abstract] OR “neurological”[Title/Abstract] OR “CNS”[Title/Abstract] OR “central nervous system”[Title/Abstract] OR “Brain tumor*”[Title/Abstract] OR “glioma*”[Title/Abstract] OR “glioblastoma”[Title/Abstract] OR “CNS lymphoma”[Title/Abstract] OR “primary CNS lymphoma”[Title/Abstract] OR “PCNSL”[Title/Abstract] OR “secondary CNS lymphoma”[Title/Abstract] OR “SCNSL”[Title/Abstract] OR “CNS involvement”[Title/Abstract] OR “autoimmune*”[Title/Abstract] OR “immune-mediated”[Title/Abstract] OR “multiple sclerosis”[Title/Abstract] OR “MS”[Title/Abstract] OR “NMOSD”[Title/Abstract] OR “neuromyelitis optica”[Title/Abstract] OR “stiff-person syndrome”[Title/Abstract] OR “myasthenia gravis”[Title/Abstract]) AND (“ICANS”[Title/Abstract] OR “immune effector cell-associated neurotoxicity syndrome”[Title/Abstract] OR “neurotoxicity”[Title/Abstract] OR “neurotoxic*”[Title/Abstract] OR “CAR-T neurotoxicity”[Title/Abstract] OR “treatment-related neurotoxicity”[Title/Abstract] OR “encephalopathy”[Title/Abstract] OR “CRES”[Title/Abstract])  Filters: Date: 2020/01/01-2025/12/10; Language: English; Species: Humans | 89 |
| Scopus | Search Terms:  TITLE-ABS-KEY (“CAR T-cell*” OR “chimeric antigen receptor T-cell*” OR “CAR-T” OR “CAR T therapy” OR “CAR-T therapy” OR “CAR T lymphocyte*” OR “CAR-T immunotherapy” OR “CAR T-cell therapy” OR “CAR T cells” OR “chimeric antigen receptor T therapy”) AND TITLE-ABS-KEY (“neurology*” OR “neurological” OR “CNS” OR “central nervous system” OR “Brain tumor*” OR “glioma*” OR “glioblastoma” OR “CNS lymphoma” OR “primary CNS lymphoma” OR “PCNSL” OR “secondary CNS lymphoma” OR “SCNSL” OR “CNS involvement” OR “autoimmune*” OR “immune-mediated” OR “multiple sclerosis” OR “MS” OR “NMOSD” OR “neuromyelitis optica” OR “stiff-person syndrome” OR “myasthenia gravis”) AND TITLE-ABS-KEY (“ICANS” OR “immune effector cell-associated neurotoxicity syndrome” OR “neurotoxicity” OR “neurotoxic*” OR “CAR-T neurotoxicity” OR “treatment-related neurotoxicity” OR “encephalopathy” OR “CRES”)  Limits: Year: 2020-2025; Document Type: Article OR Review; Language: English | 78 |
| Embase | Search Terms:  (‘CAR T CELL*’:AB,TI OR ‘CHIMERIC ANTIGEN RECEPTOR T CELL*’:AB,TI OR ‘CAR-T’:AB,TI OR ‘CAR T THERAPY’:AB,TI OR ‘CAR-T THERAPY’:AB,TI OR ‘CAR T LYMPHOCYTE*’:AB,TI OR ‘CAR-T IMMUNOTHERAPY’:AB,TI OR ‘CHIMERIC ANTIGEN RECEPTOR T THERAPY’:AB,TI OR ‘CAR T-CELL THERAPY’:AB,TI OR ‘CAR T CELLS’:AB,TI) AND (‘NEUROLOGY*’:AB,TI OR ‘NEUROLOGICAL’:AB,TI OR ‘CNS’:AB,TI OR ‘CENTRAL NERVOUS SYSTEM’:AB,TI OR ‘BRAIN TUMOR*’:AB,TI OR ‘GLIOMA*’:AB,TI OR ‘GLIOBLASTOMA’:AB,TI OR ‘CNS LYMPHOMA’:AB,TI OR ‘PRIMARY CNS LYMPHOMA’:AB,TI OR ‘PCNSL’:AB,TI OR ‘SECONDARY CNS LYMPHOMA’:AB,TI OR ‘SCNSL’:AB,TI OR ‘CNS INVOLVEMENT’:AB,TI OR ‘AUTOIMMUNE*’:AB,TI OR ‘IMMUNE-MEDIATED’:AB,TI OR ‘MULTIPLE SCLEROSIS’:AB,TI OR ‘MS’:AB,TI OR ‘NMOSD’:AB,TI OR ‘NEUROMYELITIS OPTICA’:AB,TI OR ‘STIFF-PERSON SYNDROME’:AB,TI OR ‘MYASTHENIA GRAVIS’:AB,TI) AND (‘ICANS’:AB,TI OR ‘IMMUNE EFFECTOR CELL-ASSOCIATED NEUROTOXICITY SYNDROME’:AB,TI OR ‘NEUROTOXICITY’:AB,TI OR ‘NEUROTOXIC*’:AB,TI OR ‘CAR-T NEUROTOXICITY’:AB,TI OR ‘TREATMENT-RELATED NEUROTOXICITY’:AB,TI OR ‘ENCEPHALOPATHY’:AB,TI OR ‘CRES’:AB,TI)  Limits: Year: 2020-2025; Language: English; Human Studies | 67 |
| Web of Science | **Search Terms:** TS=(“CAR T-CELL*” OR “CHIMERIC ANTIGEN RECEPTOR T-CELL*” OR “CAR-T” OR “CAR T THERAPY” OR “CAR-T THERAPY” OR “CAR T LYMPHOCYTE*” OR “CAR-T IMMUNOTHERAPY” OR “CHIMERIC ANTIGEN RECEPTOR T THERAPY” OR “CAR T-CELL THERAPY” OR “CAR T CELLS”) AND TS=(“NEUROLOGY*” OR “NEUROLOGICAL” OR “CNS” OR “CENTRAL NERVOUS SYSTEM” OR “BRAIN TUMOR*” OR “GLIOMA*” OR “GLIOBLASTOMA” OR “CNS LYMPHOMA” OR “PRIMARY CNS LYMPHOMA” OR “PCNSL” OR “AUTOIMMUNE*” OR “IMMUNE-MEDIATED” OR “MULTIPLE SCLEROSIS” OR “MS” OR “NMOSD” OR “NEUROMYELITIS OPTICA” OR “STIFF-PERSON SYNDROME” OR “MYASTHENIA GRAVIS”) AND TS=(“ICANS” OR “NEUROTOXICITY” OR “NEUROTOXIC*” OR “ENCEPHALOPATHY” OR “CRES”)  Refinements**:** Timespan: 2020-2025; Document Types: Article OR Review; Language: English | 46 |
| Total |  | 280 |

**ab,ti** = abstract and title fields**; TS** = Topic Search (title, abstract, author keywords, Keywords Plus); **ICANS =** Immune Effector Cell-Associated Neurotoxicity Syndrome; **CRES** = CAR T-Related Encephalopathy Syndrome; **CNS =** Central Nervous System; **PCNSL** = Primary Central Nervous System Lymphoma; **NMOSD =** Neuromyelitis Optica Spectrum Disorder; **MS =** Multiple Sclerosis

**Table S2.** Summary of included 33 studies

| **Author** | **Objectives** | **Country** | **Sample size** | **Study design** | **Main findings** |
| --- | --- | --- | --- | --- | --- |
| Han [6] | Assess pooled ICANS incidence for hematologic malignancies; compare by agents, diseases, and co-stimulatory domains. | Korea | 3,184 patients across 75 trials. | Systematic review | Pooled ICANS: 26.9% all-grade (95% CI 21.7–32.7%), 10.5% high-grade (95% CI 8.1–13.6%); anti-CD19 > anti-BCMA (OR 4.6 high-grade); leukemia/lymphoma > myeloma; real-world axi-cel (CD28, 54.0%/26.4%) > tisa-cel (4-1BB, 17.2%/6.1%). |
| Qin [10] | Evaluate anti-BCMA CAR T-cell (CT103A) efficacy and safety in R/R AQP4-IgG+NMOSD | China | N=12 | Phase 1 clinical trial | 11/12 relapsed-free; disability improved; seronegative for AQP4-IgG; CRS 100% Grade 1; no ICANS; Grade 3 neutropenia |
| Fischbach [11] | Demonstrate feasibility of KYV-101 in treatment-resistant MS | Germany | N=2 | Case report | Stable EDSS; reduction in intrathecal oligoclonal bands; Grade 1CRS; no ICANS observed |
| Faissner [12] | Evaluate clinical effects of a single infusion of autologous KYV-101) in a patient with severe, treatment-refractory stiff-person syndrome. | Germany | N=1 | Case report | Anti-CD19 CAR T-cells infusion achieved rapid B cell depletion & anti-GAD65 titer reduction from 1:3200 to 1:320 by day 144. Clinical efficacy: >100% walking speed increase (0.37 to 0.83 m/s), daily distance from <50m to >6km within 3 mo, and 40% GABAergic medication reduction. Stiffness improved per Modified Ashworth scale, with sustained benefits over 6 mo. |
| Chan [15] | Assess safety/feasibility of CAR T-cells in glioblastoma via 13 phase I trials | UK | 128 patients across 13 trials. | Systematic review | 44% (56/128) achieved PR/CR. Central delivery (intraventricular/intratumoral) superior to IV (higher CSF CAR T-cell, better OS *P*<0.05); median OS 2.9-14.5mo, limited by poor persistence & antigen escape; only 2 DLTs at >2.5×10^7 cells. |
| Xiao [18] | Summarize mechanism of CRS/ICANS & management strategies | China | N/A | Literature Review | CRS incidence 42-100%; ICANS 21-64%; mechanisms involve macrophage activation and cytokine pathways |
| Konitsioti [19] | Overview of CAR T-cell therapy in CNS autoimmunity | Germany | N/A | Systematic review | Limited but promising evidence; Phase I trials show safety in NMOSD; controlled trials warranted |
| Tallantyre  [20] | Summarize clinical manifestations and treatment of ICANS | UK | N/A | Literature review | ICANS occurs in >50% of recipients; median onset 4-6 days; CAR design significantly influences risk |
| Genoud [21] | Review current understanding of ICANS pathophysiology following CAR T-cell therapy | Switzerland | N/A | Literature review | ICANS pathophysiology involves endothelial activation, BBB disruption, and complex cytokine-immune cell interactions. Myeloid cells play central role. Early recognition and treatment with dexamethasone critical for favorable outcomes |
| Gust [22] | Discuss the role of cytokines in ICANS pathophysiology following CAR T-cell therapy | USA | N/A | Literature review | Cytokines, particularly IL-6, IL-1, and GM-CSF, are pivotal in ICANS development through endothelial activation & neuroinflammation. IL-1 elevation precedes IL-6 by up to 24 hours. Anakinra (IL-1 blocker) more effective than tocilizumab in preventing lethal neurotoxicity |
| Fleischer [23] | Systematically identify, classify, and clarify all previously described neuro-psychiatric AEs associated with CAR T-cell therapy | Germany | 546 studies | Systematic review | Expressive aphasia, impaired attention, and altered consciousness were most common neuropsychiatric symptoms. Early intervention with corticosteroids and tocilizumab can reduce symptom severity. Long-term sequelae observed in some patients |
| Lei [24] | Determine comprehensive rates and severity of CRS and neurological symptoms across cancer types and CAR T-cells targets | China | N=2592 (84 studies) | Systematic review+meta-analysis | Pooled rate of all-grade CRS was 77% with neurological symptoms in 40%. Higher rates of severe CRS and neurological symptoms observed in hematological malignancies. Symptom management strategies varied across studies |
| Brittain [25] | Review the current status and future perspectives of CAR T-cell therapy use in immune mediated neurological disease | UK | N/A | Literature review | CAR T-cell therapy shows promise for neurological auto-immune conditions including MS, neuromyelitis optica and MS. May provide more selective approach than hematopoietic stem cell transplantation. Long-term follow-up needed to assess durability |
| Gu [26] | Review recent findings about ICANS after CAR T-cell therapy and discuss cellular and molecular mechanisms | China | N/A | Literature review | ICANS pathogenesis involves peripheral immune over-activation, endothelial activation-induced BBB dysfunction, and subsequent CNS inflammation. Key mechanisms include monocyte-macrophage activation, cytokine release (IL-1, IL-6, IFN-γ, TNF-α, GM-CSF) and astrocyte injury |
| Möhn [27] | Determine the frequency and severity of CAR T-cell-related neurotoxicity and develop appropriate diagnostic and therapeutic approaches | Germany | N= 15 patients with DLBCL | Prospective cohort | ICANS occurred in 4/15 patients (27%). All 3 patients with grade 2 ICANS showed similar symptoms (apraxia, aphasia, hallucinations). Dexamethasone (40 mg/day) rapidly resolved symptoms in all 3 patients. Structured neurological baseline examination & close monitoring are essential for early detection |
| Qin [28] | Investigate CAR T-cell kinetics & immune alterations in NMOSD patients treated with anti-BCMA CAR-T cells using single cell multi-omics sequencing | China | N=5 patients with NMOSD | Phase I clinical trial | Proliferating cytotoxic-like CD8+ CAR T-cell clones were main effectors. Anti-BCMA CAR-T cells with enhanced chemotaxis efficiently crossed the blood-CSF barrier, eliminated plasma blasts/plasma cells, and suppressed neuroinflammation. CD44-expressing early memory CAR-T cells showed potential for persistence |
| Dong [29] | Assess safety, efficacy, and molecular mechanism of anti-BCMA CAR T-cell therapy in refractory CIDP. | China | N=2 | Case report (two cases) | Both achieved drug-free remission within 6 months; patients 1 relapsed at 12 mo post-COVID-19. Patients 2 sustained ˃ 24 mo of remission. |
| Mulvey [30] | Review novel strategies for managing CAR T-cells toxicity (CRS/ICANS) | Switzerland | N/A | Literature review | Novel engineering strategies (on-off switches, tunable CARs) reduce toxicity while maintaining efficacy |
| Wesley [31] | Characterized EEG findings during ICANS & understand EEG patterns in broader clinical context | USA | N=29 | Retrosp cohort (Single centre) | Diffuse slowing 97.2% triphasic waves 41.7%; no electrographic seizures despite clinical seizures being mortality predictor (*P*=0.008) |
| Cassanello [32] | Assess outcomes of CD 19 CAR T-cell therapy in R/R DLBCL with CNS involvement | Multinational (US, Italy, Spain) | N=54 | Retrosp cohort (multi-centre) | ORR 73% .CR 65%, median PFS 7.5 mo.; Tisa-cell showed worse outcomes vs Axi-cel |
| Gaballa [33] | Investigate safety and efficacy of BCMA-directed CAR T-cell therapy in MM patients with CNS involvement | USA | N=10 | Retrosp cohort (multi-centre) | ORR 80% CNS response 100% median PFS 6.3 mo, median OS 13.3 mo; no grade 3+ CRS; optimized bridging key |
| Bajwa [34] | Assess siltuximab efficacy for CRS and ICANS | USA (6 centers) | N=54 | retrosp cohort (multi-centre) | Siltuximab: 75% CRS response, 60% ICANS response; effective in tocilizumab-exposed CRS (63%) and steroid-exposed ICANS (53%). |
| Hegelmaier [35] | Describe first use of anti-CD 19 CAR T-cells in treatment-refractory DAGLA antibody-ssociated autoimmune encephalitis | Germany | N=1 | Case report | Clinical CR; anti-DAGLA antibodies; sustained 1-year improvement; complete B cell depletion |
| He [36] | Evaluate efficacy and safety of third-generation CD19 CAR T- cells in R/R B-cell malignancies with CNS involvement | China | N=21 | Phase II clinical trial | 71% ORR, median DOR 11.1 months; 12-month PFS 41.5%, OS 61.2% at 20.4-month median follow-up; ICANS 43% (29% grade ≥3, manageable); CSF CAR T-cell detection confirmed CNS penetration correlating with response |
| Qin [37] | Evaluate the safety, feasibility, & efficacy of anti-BCMA CAR T-cell therapy (equecabtagene autoleucel) in patients with refractory progressive MS | China | N=5 | Phase I clinical trial | All patients showed clinical improvements in EDSS, 9-HPT, and T25FW scores; 83% achieved long-term CR off medications, with complete CSF oligoclonal band clearance, kappa free light chain reductions, and no new MRI lesions (T1 gadolinium-enhancing or enlarging T2). Safety included grade 1 CRS in 80%, no ≥grade 2 CRS/ICANS, and transient grade ≥3 cytopenias within 40 days. |
| Granit [39] | Evaluate safety and preliminary clinical activity of Descartes-08 (autologous anti-BCMA rCAR T-cells) in adults with gMG) | USA | N=14 | phase Ib/IIa clinical trial | Safe/tolerable (no DLTs, CRS, ICANS, grade ≥3 AEs); mean week 12 reductions: MG-ADL 5.9 points, QMG 7, 89% repeatable improvements, 3 achieved minimal symptom expression, 2 eliminated IVIg dependence (persisting to 9 mo); modest anti-AChR titer drop (22%), sBCMA/BAFF/APRIL reductions, preserved humoral immunity. |
| Zhang [40] | Assess safety and efficacy of anti-BCMA/CD19 bispecific CAR T-cells in refractory gMG | China | N=18 | Phase I trial | 100% ≥2-point MG-ADL reduction, 100% ≥3-point QMG reduction; no ICANS; B-cell recovery 56% by day 180 |
| Ortuno‑  Sahagún [41] | Synthesize preclinical and early clinical evidence on CAR T-cells for MS; outline barriers; discuss optimization strategies for safety/targeting/scalability | Mexico | N/A | Literature article | Anti-CD19/BCMA CAR T shows CNS penetration, B-cell depletion, no ICANS/CRS in initial patients (e.g., KYV-101 NCT06138132: 4 pts); promising for progressive MS but neurotoxicity risks such as delayed parkinsonian syndromes or PML remain concerns |
| Graham [42] | Develop best practice guidelines for identifying, diagnosing, monitoring, & managing non-ICANS neurological complications post-therapy | Multinational | N/A: review | Litreture review | Non-ICANS CAR T-cell neurotoxicities: MNT (BCMA myeloma, parkinsonism-like, steroids/cyclophosphamide); CN nerve palsies (6% cilta-cel, facial, reversible steroids); TIAN (CNS tumors: ICP rise/edema or focal worsening); stroke (~1-3%, post-ICANS, urgent CTA); myelopathy (~24 cases, acute, steroids/anakinra); neuropathy/Guillain-Barré (6%, IVIg/steroids) |
| Vonberg [43] | Review neurotoxic AEs of CAR T-cell therapy, focusing on ICANS while highlighting emerging non-classical toxicities, to guide diagnosis, grading, and management. | UK | N/A: review | Litreture review | ICANS (20-70% incidence, median onset day 5, peaks day 7-8) features encephalopathy, expressive dysphasia/dysgraphia (ICE score), graded via ASTCT/ICE (low 1-2 vs high ≥3); pathophysiology involves CRS-linked BBB/endothelial disruption, emerging non-ICANS (MNT parkinsonism, CN palsies, TIAN, myelopathy, stroke <10%). |
| Lazzari  [44] | Report BCMA CAR T-cell (ciltacabtagene autoleucel) neurotoxicity manifesting as progressive cranial neuropathies | USA | N=1 | Case report | 75yo male developed left CN VII palsy (day 19 post-CAR T-cell), progressing to bilateral VII+left VI palsy (day 31); MRI: bilateral VII nerve enhancement; no ICANS by ASTCT criteria; extensive -ve workup; improved with high-dose IV steroids+prolonged taper (CN VI resolved 2.5 mo, facial HB II/III at 5.5mo). |
| Patrick [45] | Report bilateral VII nerve palsy as novel CAR T-cell neurotoxicity in multiple myeloma | Canada | N=1 | Case report | Acute bilateral VII palsy 2 weeks post-BCMA CAR T-cell (ciltacabtagene autoleucel) after grade 1 CRS; MRI mild VII enhancement; CSF, -ve infectious/cytology workup; no ICANS (ICE=10); full resolution by day +42 after dexamethasone |
| Gómez-Llobell (2025) [46] | Validate ICANS risk models using serum/CSF cytokines in CD19 CAR T-cell recipients | Spain | N=101 | Retrosp cohort (multi-centre) | CSF cytokines (IL-6, IL-15) correlated with grade 2–4 ICANS; models AUC 0.80-0.84; persistent CSF elevation tracked resolution. |

**AEs**: Adverse effects, **AQP4-IgG**: Aquaporin-4 immunoglobulin G, **ASTCT**: American Society for Transplantation and Cellular Therapy, **axi-cel**: Axicabtagene ciloleucel, **BBB**: Blood-brain barrier, **BCMA**: B-cell maturation antigen, **CAR T-cell**: Chimeric antigen receptor T-cell, **CD19**: Cluster of differentiation 19, **CD8**: Cluster of differentiation 8, **CD44**: Cluster of differentiation 44, CI: Confidence interval, **CIDP**: Chronic inflammatory demyelinating polyneuropathy, cilta-cel: Ciltacabtagene autoleucel, **CN**: Cranial nerve, **CNS**: Central nervous system, **CR**: Complete responses, **CRS**: Cytokine release syndrome, **CSF**: Cerebrospinal fluid, **CT103A**: BCMA-targeted CAR T-cell product, **DLBCL**: Diffuse large B-cell lymphoma, **DLTs**: Dose-limiting toxicities, **DOR**: Duration of response, **EDSS**: Expanded Disability Status Scale, **EEG**: Electroencephalogram, **GABAergic**: Gamma-aminobutyric acid-ergic, **gMG**: Generalized myasthenia gravis, **GM-CSF**: Granulocyte-macrophage colony-stimulating factor, **HB**: House-Brackmann, **9-HPT**: Nine-Hole Peg Test, **ICE**: Immune Effector Cell-associated Encephalopathy, **ICANS**: Immune effector cell-associated neurotoxicity syndrome, **ICP**: Intracranial pressure, **IV**: Intravenous, **IVIg**: Intravenous immunoglobulin, **KYV-101**: Anti-CD19 CAR T-cell product, **MG-ADL**: Myasthenia Gravis-Activities of Daily Living, **MM**: Multiple myeloma, **MNT**: Movement and neurocognitive toxicity, **MRI**: Magnetic resonance imaging, **MS**: Multiple sclerosis, **NA**: Not applicable, **NMOSD**: Neuromyelitis optica spectrum disorder, **ORR**: Objective response rate, **OS**: Overall survival, **PFS**: Progression-free survival, **PML**: Progressive multifocal leukoencephalopathy, **PR**: partial responses**, QMG:** Quantitative Myasthenia Gravis, **rCAR-T:** Regulatory CAR T-cell, **Retrosp:** Retrospective, **R/R**: Relapsed/refractory, **T25FW**: Timed 25-Foot Walk, **TIAN**: Tumor inflammation-associated neurotoxicity, **tisa-cel**: Tisagenlecleucel, **-ve:** negative**, yo:** year-old.

**Table S3**. Preferred Reporting Items for Systematic reviews and Meta-Analyses extension for Scoping Reviews (PRISMA-ScR) Checklist

| **SECTION** | **ITEM** | **PRISMA-ScR CHECKLIST ITEM** | **REPORTED ON PAGE #** |
| --- | --- | --- | --- |
| **TITLE** | | | |
| Title | 1 | Identify the report as a scoping review. | Page 1 |
| **ABSTRACT** | | | |
| Structured summary | 2 | Provide a structured summary that includes (as applicable): background, objectives, eligibility criteria, sources of evidence, charting methods, results, and conclusions that relate to the review questions and objectives. | Page 1 |
| **INTRODUCTION** | | | |
| Rationale | 3 | Describe the rationale for the review in the context of what is already known. Explain why the review questions/objectives lend themselves to a scoping review approach. | Page 1-2 |
| Objectives | 4 | Provide an explicit statement of the questions and objectives being addressed with reference to their key elements (e.g., population or participants, concepts, and context) or other relevant key elements used to conceptualize the review questions and/or objectives. | Page 2 |
| **METHODS** | | | |
| Protocol and registration | 5 | Indicate whether a review protocol exists; state if and where it can be accessed (e.g., a Web address); and if available, provide registration information, including the registration number. | Page 2 |
| Eligibility criteria | 6 | Specify characteristics of the sources of evidence used as eligibility criteria (e.g., years considered, language, and publication status), and provide a rationale. | Page 2 |
| Information sources* | 7 | Describe all information sources in the search (e.g., databases with dates of coverage and contact with authors to identify additional sources), as well as the date the most recent search was executed. | Page 2 |
| Search | 8 | Present the full electronic search strategy for at least 1 database, including any limits used, such that it could be repeated. | Page 2 |
| Selection of sources of evidence† | 9 | State the process for selecting sources of evidence (i.e., screening and eligibility) included in the scoping review. | Page 2 |
| Data charting process‡ | 10 | Describe the methods of charting data from the included sources of evidence (e.g., calibrated forms or forms that have been tested by the team before their use, and whether data charting was done independently or in duplicate) and any processes for obtaining and confirming data from investigators. | Page 2-3 |
| Data items | 11 | List and define all variables for which data were sought and any assumptions and simplifications made. | Page 2-3 |
| Critical appraisal of individual sources of evidence§ | 12 | If done, provide a rationale for conducting a critical appraisal of included sources of evidence; describe the methods used and how this information was used in any data synthesis (if appropriate). | Page 2 |
| Synthesis of results | 13 | Describe the methods of handling and summarizing the data that were charted. | Page 3 |
| **RESULTS** | | | |
| Selection of sources of evidence | 14 | Give numbers of sources of evidence screened, assessed for eligibility, and included in the review, with reasons for exclusions at each stage, ideally using a flow diagram. | Page 2 |
| Characteristics of sources of evidence | 15 | For each source of evidence, present characteristics for which data were charted and provide the citations. | Page 3 |
| Critical appraisal within sources of evidence | 16 | If done, present data on critical appraisal of included sources of evidence (see item 12). | Page 2 |
| Results of individual sources of evidence | 17 | For each included source of evidence, present the relevant data that were charted that relate to the review questions and objectives. | Page 3-7 |
| Synthesis of results | 18 | Summarize and/or present the charting results as they relate to the review questions and objectives. | Page 3–7 |
| **DISCUSSION** | | | |
| Summary of evidence | 19 | Summarize the main results (including an overview of concepts, themes, and types of evidence available), link to the review questions and objectives, and consider the relevance to key groups. | Page 7 |
| Limitations | 20 | Discuss the limitations of the scoping review process. | Page 7 |
| Conclusions | 21 | Provide a general interpretation of the results with respect to the review questions and objectives, as well as potential implications and/or next steps. | Page 7-8 |
| **FUNDING** | | | |
| Funding | 22 | Describe sources of funding for the included sources of evidence, as well as sources of funding for the scoping review. Describe the role of the funders of the scoping review. | Page 8 |
